# Ovarian malignancy unmasked by venous gangrene in a patient on warfarin therapy: a case report

**DOI:** 10.1186/s13256-016-0939-y

**Published:** 2016-06-06

**Authors:** Amgad Said, Justin Keasberry

**Affiliations:** Internal Medicine department at Princess Alexandra Hospital, University of Queensland, Brisbane, Australia

**Keywords:** Venous gangrene, Thrombosis, Ovarian, Malignancy, Warfarin, VKA, Vitamin K antagonists

## Abstract

**Background:**

Venous gangrene complicating deep vein thrombosis in the context of anticoagulation use in patients with gynecologic malignancy is rarely reported. We report an unusual presentation of venous gangrene of the lower limbs associated with warfarin therapy in a 53-year-old woman from the Cook Islands with an occult ovarian cancer.

**Case presentation:**

A 53-year-old woman of Cook Islands origin presented with exertional dyspnea, rapid atrial fibrillation, bilateral lower limb edema, and painful digital ischemia of her hallux. She was on warfarin therapy for atrial fibrillation and had a stable therapeutic international normalized ratio. Bilateral proximal lower limb deep vein thrombosis and digital gangrene subsequently developed in the setting of a supratherapeutic international normalized ratio and platelet count depletion. Her warfarin was reversed and heparin therapy was commenced with resulting correction of her thrombocytopenia.

**Conclusions:**

We would like to attract the attention of the reader to the potential hazard of the use of warfarin in patients with malignancy. In our case, we also demonstrated a predictive value of supratherapeutic international normalized ratio and platelet count depletion that could herald massive thrombosis and gangrene in a patient who was previously stable on warfarin therapy. Early recognition and prompt reversal of warfarin in these circumstances is essential to correct the unbalanced prothrombotic process that leads to extensive thrombosis and gangrene. The outlook of such cases remains dismal and results in extensive morbidity and mortality.

## Background

Venous gangrene complicating deep vein thrombosis (DVT) in the context of anticoagulation use in patients with gynecological malignancy is rarely reported [1]. The diagnosis can be delayed if not suspected due to its rare occurrence and invariably result in a fatal outcome [2]. This case report documents a 53-year-old woman who developed bilateral lower limb DVT while being on a therapeutic dose of warfarin for atrial fibrillation (AF) which rapidly progressed to venous gangrene of both feet and subsequently was discovered to have ovarian cancer.

## Case presentation

A 53-year-old morbidly obese woman with a body mass index (BMI) of 70 of Cook Islands origin had a background of obesity-induced hypoventilation and metabolic syndrome, chronic kidney disease, and gout. She also had ischemic cardiomyopathy and received oral anticoagulation with warfarin for AF. She had a stable international normalized ratio (INR) for 8 years prior to her presentation. She complained of constitutional symptoms, weight loss, fatigue, and mild abdominal discomfort and distension in the 4 months prior to her presentation. She was admitted due to ongoing exertional dyspnea, bilateral painful lower limb edema, and rapid AF.

**Fig. 1:**
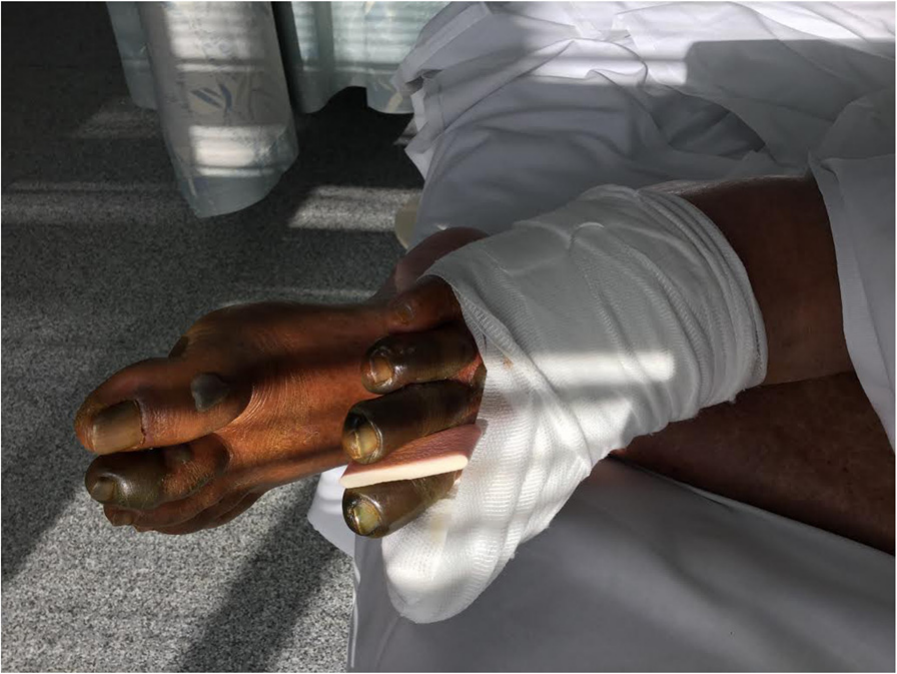
Photograph revealing the extent of digital ischemia with a bandaged right hallux due to serous wound discharge

On her second day in hospital, she developed worsening ischemia of her first, second and third toes of her right foot and second and third toes of her left foot. Her toes became dusky and turned black in color and more extensive discoloration was noted on the right side (Fig. 1). Pulsations remained intact and arterial Doppler ultrasound of her lower limbs failed to demonstrate significant arterial disease. Distal vascular disease was thought to be responsible for her toe gangrene. Her warfarin was still continued at this point.

**Fig. 2:**
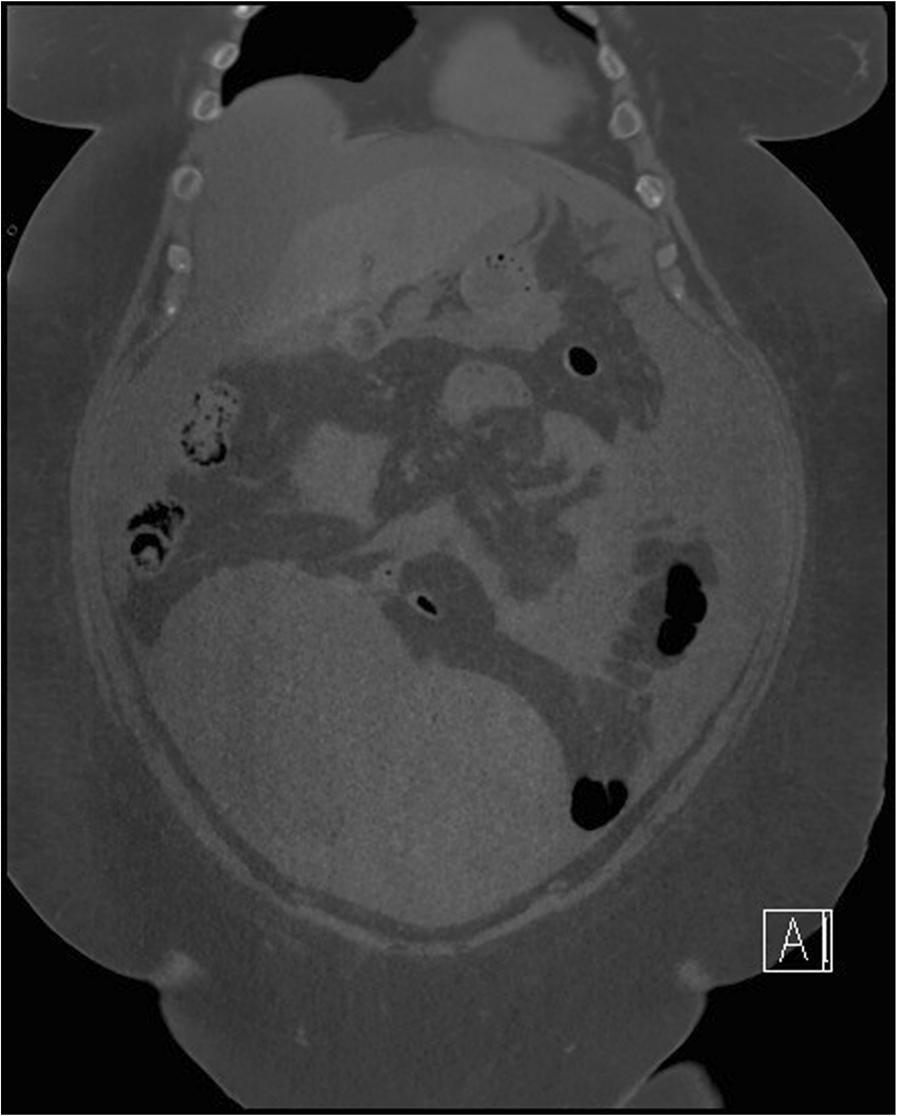
Non-contrast abdominal computed tomography of the patient revealing the 18-cm large ovarian lesion with moderate volume peritoneal ascites

A computed tomography (CT) scan of her abdomen (Fig. 2) and pelvis revealed an 18-cm cystic ovarian mass and free abdominal ascites. An ultrasound-guided ascitic tap was negative for malignant cells and her serum to ascites albumin gradient was 12 g/L. Subsequently, her plasma levels of cancer antigen-125 (CA-125) were 3410 u/ml (normal <35), human epididymis protein 4 (HE4) was 30,300 pmol/L (normal <70) and Risk of Ovarian Malignancy Algorithm (ROMA) was 100 %. Values of ROMA >25.3 % in postmenopausal women indicate high risk of malignancy with >95 % sensitivity and >76 % specificity [3].

On the third day, her INR progressively increased to 4.5 and simultaneously her platelet count decreased from 154 to 108×10^9^/L. At the same time, serial activated partial thromboplastin time (aPTT) measurements remained normal at 26 to 36 seconds and the derived fibrinogen levels also remained normal on serial measurements 3.8 g/L (1.7 to 4.5 g/L). No red cell fragments were seen on repeat blood film. The result of a qualitative D-dimer test was weakly positive.

**Fig. 3:**
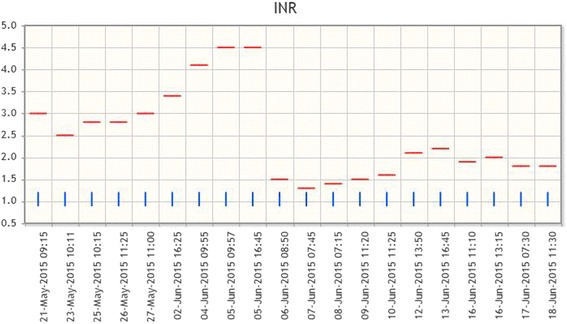
International normalized ratio results. International normalized ratio results on *y*-axis; date of result on *x*-axis. Note warfarin was ceased and reversed on 5 June 2015. The red bars represent INR results above the normal reference range which is represented by the blue bars. *INR* international normalized ratio

With the aforementioned results, coagulopathy related to both warfarin and an underlying ovarian cancer was suspected. Her anticoagulation with warfarin was reversed with vitamin K as evidenced by the drop in her INR (Fig. 3) and switched to an unfractionated heparin administered intravenous infusion. A subsequent venous Doppler confirmed extensive bilateral lower limb DVT involving all deep veins on her left and an acute thrombosis of the popliteal vein on her right side.

**Fig. 4:**
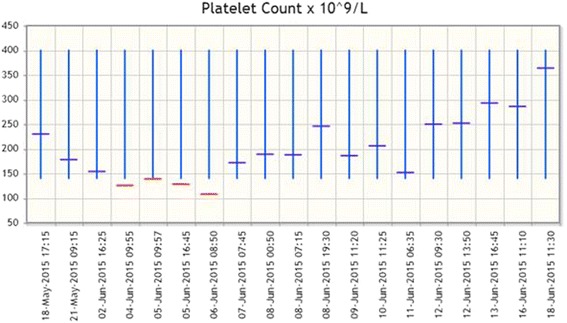
Platelet count results. Platelet count results on *y*-axis; date of result on *x*-axis. Note warfarin was ceased and reversed on 5 June 2015

We noted that her platelet count increased immediately after administration of vitamin K and intravenously administered heparin and peaked to 246×10^9^/L on the following day (Fig. 4). Because of her severe renal impairment, the use of either low molecular weight heparin or direct oral anticoagulants was precluded. She was continued on an intravenous infusion of heparin for the subsequent 2 weeks.

Serology revealed positive hepatitis B surface antigen, positive qualitative hepatitis B virus (HBV)-DNA, positive core-HBV (total antibodies, negative immunoglobulin M antibodies) and envelope-HBV antibodies suggestive of chronic hepatitis B infection. Her serum and plasma cryoglobulins, anti-beta 2 glycoprotein, and anti-cardiolipin (immunoglobulin G) antibodies were negative. Her protein C (PC) and factor VII levels were not measured since she was on chronic warfarin therapy. Her thrombin-antithrombin complexes (TAT) were not measured.

An associated oliguric acute kidney injury was managed conservatively and an ultrasound Doppler of her renal vein and renal artery ruled out thrombosis. Her estimated glomerular filtration rate (eGFR) was stable at around 20 mL/minute/1.73 m^2^.

Our patient was deemed to be at an extremely high perioperative risk for a debulking laparotomy and was deemed not suitable for surgery, thus a histological specimen was not pursued. In addition, her Eastern Cooperative Oncology Group (ECOG) functional state score of 4 and comorbidities excluded her from chemotherapy.

Eventually, due to her sepsis associated with venous gangrene of both feet, coagulopathy, gynecological malignancy, cardiomyopathy, and end-stage renal failure, palliative treatment was offered and she died 19 days after admission.

## Discussion

The development of ovarian cancer in our patient while being on warfarin is suspected to be the inciting event to our patient’s coagulopathic state and the development of venous gangrene. The presence of highly positive HE4, CA-125 and ROMA scores along with a large pelvic mass makes the diagnosis of ovarian malignancy most likely despite the negative ascitic cytology. It is likely that our patient had chronic disseminated intravascular coagulation (DIC; compensated DIC) in lieu of the ovarian cancer. This is a known precipitant of venous thromboembolism (VTE) in patients with cancer [4]. However, it is interesting that while being on a therapeutic dose of warfarin, she developed extensive thromboembolic complications.

We excluded antiphospholipid syndrome or associated vasculitis and deemed that our patient was unlikely to have a primary thrombotic state because she had no family history or personal history of thrombotic tendencies and was not on any attributable medications except warfarin. As a limitation to our approach, a histological sample of the mass was not obtained. This would have confirmed the subtype of ovarian cancer and added to the strength of association in our case report.

Warfarin is a commonly used anticoagulant. The anticoagulant properties of warfarin are attributed to the suppression of vitamin K-dependent procoagulant factors: prothrombin (factor II), factor X, factor IX and factor VII. On the other hand, the prothrombotic and coagulant properties of warfarin may result from the unbalanced suppression of the vitamin K-dependent natural anticoagulants: PC and protein S [5]. This may occur upon initiation of warfarin therapy and is attributed to the earlier depletion of the anticoagulant factors due to their shorter half-life resulting in a prothrombotic state [3].

This exceptional situation of warfarin-induced unbalanced suppression of anticoagulant factors has also been described in patients with cancer [5, 6]. In these patients there is direct activation of their clotting system by cancer cells, and indirectly by activation of platelets, monocytes and endothelial cells. Cytokines such as tumor necrosis factor-α and interleukin-1β released by tumor cells activate the tissue factor (TF) [2].

Despite depletion of factor VII in patients treated with warfarin, patients with cancer may continue to have uncontrolled activation of coagulation which is reflected by markedly increased levels of TAT complexes. This procoagulant activity is also enhanced by failure of PC to down-regulate thrombin generation [1].

It was found that a supratherapeutic INR (≥6.0), warfarin-induced PC and protein S deficiency, obesity, and metabolic syndrome were among several factors to predispose to venous thrombosis and gangrene in patients with cancer [2].

A similar outcome of venous thrombosis and gangrene that results from warfarin therapy has been described in patients with antiphospholipid antibody syndrome and in patients with heparin-induced thrombocytopenia (HIT) [2, 5]. In patients with HIT, activation of the thrombotic pathway occurs due to an antibody-mediated process. Immunoglobulin G antibodies bind to the platelet factor 4 (PF4)-heparin complexes. The resulting immune complexes crosslink receptors on platelets and monocytes thus activating them. The activation of platelets and monocytes increases thrombin generation with subsequent thrombocytopenia due to consumptive coagulopathy. Patients with established HIT may develop venous limb gangrene if warfarin is commenced due to the suppression of factor C and S that occurs upon initiation of warfarin therapy [7]. In the case of antiphospholipid antibody syndrome, initiation of warfarin therapy can lead to thromboembolism and venous gangrene by similarly suppressing the levels of PC and protein S on top of a preexisting hypercoagulable state [5].

Warfarin-induced gangrene may present as the classic picture of coumarin skin necrosis with involvement of the dermal and sub-dermal tissues predominantly at central (non-acral) sites such as the breast, abdomen, thigh, calf, and forearm [1]. This is particularly described in patients with heterozygous PC deficiency [2]. On the other hand, venous limb gangrene complicating warfarin therapy presents with digital (acral) necrosis in association with DVT. Typically, the arterial circulation remains patent with thrombosis involving both the larger veins and the smaller venules and microcirculation [1]. Of interest, in our patient the extent of digital gangrene due to occlusion of the microcirculation did not correlate to the degree of thrombosis of the larger veins on the same side.

Phlegmasia cerulea dolens, on the other hand, is a rare form of massive proximal venous thrombosis of the lower limbs which is often associated with arterial insufficiency and absent arterial pulsations. It may progress to venous gangrene and compartment syndrome and has been described in patients with particularly thrombogenic cancers including gastric, esophageal, lung, pancreatic, renal, and ovarian cancers, as well as acute myelogenous leukemia and non-Hodgkin’s lymphoma [8].

In a recent article published by Warkentin *et al*. in May 2015 [1], a novel and clinically distinct syndrome of severe venous limb gangrene (phlegmasia/venous limb gangrene) in ten patients with metastatic cancer was described. This group of patients was under treatment for DVT with heparin bridging and warfarin. They developed a rise in platelet count upon initiation of heparin therapy, which was followed by a drop in the count when heparin was discontinued. They also showed supratherapeutic INR levels upon commencement of warfarin therapy. The authors demonstrated an elevated TAT to PC activity ratio reflecting an underlying disturbed procoagulant–anticoagulant balance [1].

Our patient had previously maintained a stable therapeutic INR level with warfarin therapy. The occurrence of venous gangrene was heralded by prolongation of INR and progressive thrombocytopenia. Thromboembolism developing in patients while on warfarin therapy may either be due to a sub-therapeutic INR or due to a new procoagulant state. In this context, prolongation of INR and thrombocytopenia indicates an ongoing consumptive coagulopathy. In our patient this state was most likely perpetuated by the ovarian cancer. Warfarin depletes PC levels and in patients with cancer with enhanced thrombin generation, a prothrombotic state is established with development of microvascular thrombosis [2]. After administering vitamin K and commencement of heparin therapy, thrombocytopenia was corrected. This indicated interruption of underlying cancer-mediated thrombosis and repletion of PC and protein S.

In patients maintained on warfarin therapy, an estimation of PC and factor VII levels would be unhelpful in the diagnosis of ongoing consumptive coagulopathy. Warkentin *et al*. suggested using the ratio of TAT to PC levels to demonstrate the procoagulant–anticoagulant imbalance [1]. In our scenario, the dropping platelet count coupled with a rising INR was taken as a surrogate marker for the hyperactive prothrombotic pathway.

## Conclusions

This case demonstrates that the association of new onset thrombocytopenia when coupled with INR prolongation during warfarin therapy may herald massive thrombosis and should alert the clinician to an underlying consumptive coagulopathy. If suspected, evidence for an activated thrombotic process should be sought. In patients treated with warfarin therapy an estimation of PC and factor VII levels would be unhelpful but TAT levels may be more useful. Warfarin therapy in this situation should be reversed and a suitable alternative anticoagulant agent should be considered.

## References

[1] Warkentin TE, Cook R, Sarode R, et al. Warfarin-induced venous limb ischemia/gangrene complicating cancer: A novel and clinically distinct syndrome. Blood. 2015;126:486–93. 10.1182/blood-2015-01-622787 25979950

[2] Osman K, Ahmed M, Abdulla S, et al. Venous gangrene and cancer: A cool look at a burning issue. Int Semin Surg Oncol. 2007;4:7. doi:10.1186/1477-7800-4-7. PMC1851969 17386114

[3] Moore RG, Brown AK, Miller MC, et al. The use of multiple novel tumor biomarkers for the detection of ovarian carcinoma in patients with a pelvic mass. Gynecol Oncol. 2008;108:402–8. 10.1016/j.ygyno.2007.10.017 18061248

[4] Feinstein D. Disseminated intravascular coagulation in patients with solid tumors. Oncol J. 2015;29(2):96–102. 25683828

[5] Hostetler SG, Sopkovich J, Dean S, Zirwas M. Warfarin-induced venous limb gangrene. J Clin Aesthetic Dermatol. 2012;5(11):38–42. PMC3509881 23198012

[6] Georgin S, Pouchot J, Raschilas F, et al. Paradoxical venous limb gangrene complicating oral anticoagulation in a patient with cancer-associated deep venous thrombosis. Arch Dermatol. 2003;139:1126–8. 10.1001/archderm.139.9.1126 12975152

[7] Arepali GM, Ortel TL. Heparin-induced thrombocytopenia. N Engl J Med. 2006;355:809–17. 10.1056/NEJMcp052967 16928996

[8] Gibson JC, Britton KA, Miller AL, Loscalzo J. Out of the blue. N Engl J Med. 2014;370:1742–8. 10.1056/NEJMcps1213589 24785210

